# Differences in *SMN1* allele frequencies among ethnic groups within North America

**DOI:** 10.1136/jmg.2009.066969

**Published:** 2009-07-21

**Authors:** B C Hendrickson, C Donohoe, V R Akmaev, E A Sugarman, P Labrousse, L Boguslavskiy, K Flynn, E M Rohlfs, A Walker, B Allitto, C Sears, T Scholl

**Affiliations:** 1 Genzyme Genetics, Westborough, Massachusetts, USA; 2 Department of Hematology and Oncology, Children’s Hospital, Boston, Massachusetts, USA; 3 Broad Institute, Cancer Program, Cambridge, Massachusetts, USA

## Abstract

**Background:**

Spinal muscular atrophy (SMA) is the most common inherited lethal disease of children. Various genetic deletions involving the bi-allelic loss of *SMN1* exon 7 are reported to account for 94% of affected individuals. Published literature places the carrier frequency for *SMN1* mutations between 1 in 25 and 1 in 50 in the general population. Although SMA is considered to be a pan-ethnic disease, carrier frequencies for many ethnicities, including most ethnic groups in North America, are unknown.

**Objectives and methods:**

To provide an accurate assessment of *SMN1* mutation carrier frequencies in African American, Ashkenazi Jewish, Asian, Caucasian, and Hispanic populations, more than 1000 specimens in each ethnic group were tested using a clinically validated, quantitative real-time polymerase chain reaction (PCR) assay that measures exon 7 copy number.

**Results:**

The observed one-copy genotype frequency was 1 in 37 (2.7%) in Caucasian, 1 in 46 (2.2%) in Ashkenazi Jew, 1 in 56 (1.8%) in Asian, 1 in 91 (1.1%) in African American, and 1 in 125 (0.8%) in Hispanic specimens. Additionally, an unusually high frequency of alleles with multiple copies of *SMN1* was identified in the African American group (27% compared to 3.3–8.1%). This latter finding has clinical implications for providing accurate adjusted genetic risk assessments to the African American population.

**Conclusions:**

Differences in the frequency of SMA carriers were significant among several ethnic groups. This study provides an accurate assessment of allele frequencies and estimates of adjusted genetic risk that were previously unavailable to clinicians and patients considering testing.

**Key points**

- Precise allele frequencies for *SMN1* mutations were obtained for five North American ethnic groups by evaluating more than 5000 specimens.
- Significant differences in *SMN1* mutation frequencies were found between several populations.
- Approximately 27% of alleles in the African American specimens had two or more *SMN1* copies. This is considerably higher than other populations and results in a SMA carrier adjusted risk estimate that is five times greater than that of the Caucasian population.
- This study provides accurate estimates of allele frequency and adjusted genetic risk for five major ethnic groups in North America.

With an incidence of 1 in 6000 to 1 in 10 000 live births, spinal muscular atrophy (SMA) is the most common lethal genetic disease of children.^1^ SMA is a neuromuscular disorder that leads to progressive, proximal muscle weakness and atrophy. Sixty per cent of individuals affected with SMA have the most severe form, type I, also known as Werdnig–Hoffman disease (Online Mendelian Inheritance in Man (OMIM) 253300), with clinical onset usually occurring before 6 months and death by respiratory failure before the age of 2 years. Clinical onset of type II SMA (OMIM 253550) usually occurs by the age of 15 months with most cases surviving beyond 10 years of age. Type II patients may learn to sit, but never gain the ability to walk. Type III SMA (OMIM 253400) is a milder form with onset typically occurring between 2 and 17 years of age. An adult form of SMA (type IV, OMIM 271150) has also been described.

SMA is caused by mutations in the Survival Motor Neuron 1 (*SMN1*) gene.^2^ The *SMN1* gene resides in a duplicated region of chromosome 5q13 telomeric to the near identical homologue *SMN2*. More than 90% of the functional SMN protein is contributed by *SMN1*. Comparably *SMN2* produces only a small fraction of SMN due to a point mutation in exon 7 that disrupts an exon splice enhancer site, preventing normal post-transcription processing.^3^ ^4^ In the general population the copy number of *SMN1* and *SMN2* genes varies. Published information suggests that 85–95% of the population has one copy of the *SMN1* gene per allele; however, individuals with more than one copy per allele have been identified.^5^ ^6^ ^7^ ^8^ ^9^ ^10^ Ninety-four per cent of affected individuals possess bi-allelic mutations involving various deletion mutations that share the loss of *SMN1* exon 7. Of the remaining 6% of SMA cases, most are attributed to a variety of rare molecular lesions distributed throughout the gene, including point mutations, small insertions, and deletions. A minority of cases may involve genes other than *SMN1*.^11^ Additionally, there is an inverse correlation between *SMN2* copy number and disease severity, such that affected individuals with three or more copies of *SMN2* typically have milder forms of the disease.^12^ This mutation spectrum permits sensitive detection of carriers through the analysis of *SMN1* exon 7 copy number by quantitative polymerase chain reaction (PCR).^5^ ^13^ ^14^ In calculating the mean from the results of their own and other published research, Ogino *et al* reported a frequency for one-copy carriers at approximately 1 in 40 individuals in the general population.^15^ In addition to the low prevalence mutations distributed across the gene, another small subset of carriers cannot be identified by exon 7 copy measurement since they have one allele with two *SMN1* copies paired with a zero-copy allele (hereafter referred to as “2+0” genotype). By exon 7 quantification these individuals are indistinguishable from wild-type (or “1+1” genotype). It has been estimated that approximately 1 in 600 apparent two-copy specimens may be “2+0” carriers.^16^

Recently published guidelines from the American College of Medical Genetics (ACMG) recommend universal carrier screening for SMA.^17^ Before this guidance, carrier screening in the USA was offered primarily to family members of individuals diagnosed with SMA. SMA is considered to be pan-ethnic; however, published studies to date have involved small and heterogeneous sample sets.^5^ ^6^ ^7^ ^8^ ^15^ ^16^ Even the few studies addressing specific races provide insufficient information to support the calculation of carrier frequencies and risk assessments with the precision warranted by widespread carrier screening.^14^ ^18^ ^19^ ^20^

To address this need for accurate allele frequency data, more than 1000 samples from five major ethnic groups, African American, Ashkenazi Jewish, Asian, Caucasian and Hispanic, were tested for *SMN1* copy number. These ethnicities are relevant since they comprise >95% of the North American population at large and the North American patients electing to participate in genetic testing (2006 US Census estimates).

## Methods

Samples were collected from residual material following routine clinical testing of individuals presumed to have no family history of SMA. All specimens were made completely anonymous before testing in accordance with approved institutional protocols. Ethnic assignment relied upon patient reported data that were not collected as part of complete family histories. However, these ethnic assignments, which reflect clinical practice, are therefore highly representative of the anticipated clinical experience for SMA carrier screening. The assessment of *SMN1* exon 7 copy number employed a clinically validated, real-time, quantitative PCR assay specific for the single nucleotide change in exon 7 (cd 840 c>t). All reported results could be assigned to validated, non-overlapping genotype groups of 1, 2 or 3 *SMN1* copies (the three copies group includes samples with three or more *SMN1* gene copies).

## Results

Significant differences in the frequencies of *SMN1* genotypes were observed among several ethnicities (table 1). The highest one-copy carrier rate was identified in specimens from the Caucasian group with a frequency of 1 in 37 samples (2.7%). This result agrees closely with those previously reporting on European populations.^6^ ^7^ The 1.8% carrier frequency detected in the Asian sample group is comparable to the 1.6% and 1.9% that have been reported in Southern Chinese populations.^14^ ^20^ The African American and Hispanic groups had statistically significant lower one-copy carrier genotypes when compared to Caucasians, at 1 in 90 (1.1%, p = 0.0089) and 1 in 125 (0.8%, p = 0.0007), respectively. These are the lowest *SMN1* carrier frequencies reported for any population or ethnic group at this time. For all but the African American group, the two-copy genotype was more than five times more prevalent than the three-copy genotype group. This result is consistent with all previously published data showing the two-copy genotype to be predominant. Surprisingly, the African American population departed significantly from this genotype distribution, revealing similar frequencies for the two- and three-copy genotypes (52.1% and 46.8% respectively), suggesting a much higher frequency of alleles with two or more *SMN1* copies than the other four ethnic groups. These unexpected results in this group were confirmed by retesting 10% of the samples using multiplexed ligation dependant probe amplification (MLPA, MRC Holland, The Netherlands) as an alternate technique. All MLPA results were concordant with the original real-time PCR data.

**Table 1:** Frequency of *SMN1* copy number across various ethnicities

Ethnicity	1 copy		2 copies		3 copies		Total n
	n	Frequency (95% CI)	n	Frequency (95% CI)	n	Frequency (95% CI)	
Caucasian	28	0.027 (0.019 to 0.039)	935	0.910 (0.89 to 0.93)	65	0.063 (0.05 to 0.08)	1028
Ashkenazi Jewish	22	0.022 (0.015 to 0.033)	827	0.825 (0.80 to 0.85)	153	0.153 (0.13 to 0.18)	1002
Asian	18	0.018 (0.011 to 0.028)	897	0.873 (0.85 to 0.89)	112	0.109 (0.09 to 0.13)	1027
African American	11	0.011 (0.006 to 0.019)	529	0.521 (0.49 to 0.55)	475	0.468 (0.44 to 0.50)	1015
Hispanic	8	0.008 (0.004 to 0.015)	870	0.845 (0.82 to 0.87)	152	0.148 (0.13 to 0.17)	1030

Using the observed genotype data and assuming Hardy–Weinberg equilibrium, maximum likelihood estimation was employed to determine frequencies for alleles and allele pairings within all sample groups (tables 2 and 3). These calculations reveal that the expected frequency of alleles with two or more copies of *SMN1* in the African American group is 3.4 to 8.4 times more prevalent when compared to the other ethnic groups. The preponderance of the two-copy allele in the African American group also suggests a much higher frequency of individuals with the SMA carrier “2+0” genotype compared to other ancestries.

**Table 2:** Frequencies of *SMN1* copies per allele for each ethnic group

Ethnicity	0	1	2	1^D^*
Caucasian	0.0142	0.9532	0.0318	0.0003
Ashkenazi Jewish	0.0121	0.9072	0.0825	0.0002
Asian	0.0096	0.9338	0.0571	0.0002
African American	0.0077	0.7188	0.2691	0.0001
Hispanic	0.0044	0.9188	0.0804	0.0001

*1^D^ = disease allele (not caused by exon 7 deletions—for example, point mutations) as described by and based on frequency in SMA patients by Wirth *et al* (1999).^11^ De-novo mutations have also been described in spinal muscular atrophy (SMA) patients; however, their frequency is sufficiently low (∼2%) such that their inclusion in the calculations causes no change to these results at the level of precision used here.^21^

**Table 3:** Frequency of *SMN1* allele pairings

Allele pairings	Caucasian	Ashkenazi Jewish	Asian	African American	Hispanic
Non-carrier					
2+2	0.0011	0.0065	0.0032	0.0748	0.0059
2+1	0.0621	0.1462	0.1058	0.3931	0.1416
1+1	0.9081	0.8230	0.8720	0.5169	0.8439
Total	0.9713	0.9756	0.9810	0.9848	0.9914
Carrier					
2+1^D^*	1.7E–05	3.6E–05	2.0E–05	7.7E–05	1.2E–05
2+0	0.0009	0.0019	0.0011	0.0041	0.0007
1+1^D^	0.0005	0.0004	0.0003	0.0002	0.0001
1+0	0.0272	0.0220	0.0175	0.0108	0.0078
Total	0.0286	0.0243	0.0189	0.0152	0.0086
Affected					
1^D^+1^D^*	7.1E–08	5.1E–08	2.0E–08	3.1E–08	6.2E–09
1^D^+0*	7.6E–06	5.5E–06	2.1E–06	3.3E–06	6.7E–07
0+0	2.0E–04	1.5E–04	5.7E–05	8.8E–05	1.8E–05
Total	2.1E–04	1.5E–04	5.9E–05	9.1E–05	1.9E–05

*These genotype frequency estimates lack precision because of the limited data for the 1^D^ allele frequency.^11^

Adjusted carrier risk assessment estimates have been previously published based on aggregate results of several studies.^15^ ^16^ These estimates calculate the probability of being an SMA carrier when an individual without a family history of SMA receives a test result showing two or more *SMN1* copies. These calculations account for rare mutations undetectable by the method described here, and model the “2+0” carrier genotype. Adjusted carrier risk was calculated for each ethnic group based on its unique allele frequencies (table 4). The result for the Caucasian population (1:632) is similar to that previously reported by Smith *et al* (1:648).^16^ In that report calculations were based on data from a compilation of studies from countries in Europe, USA, and Australia. The adjusted risk estimate in the Ashkenazi Jewish population (1:350) is approximately two times higher than the Caucasian group because of the higher frequency of two-copy alleles. As expected, the unusually high frequency of two-copy alleles in the African American population produces an adjusted risk factor more than five times greater than that of Caucasians or Asians. Additionally, a “3+0” genotype carrier has been previously inferred but not been formally demonstrated by molecular techniques.^15^ The adjusted carrier risk calculations for the three-copy genotype are presented in the right hand column of table 4. Finally, using these calculated frequencies, the negative predictive value and detection rate were determined for real-time PCR quantification of exon 7 as a screening method for each ethnic group (table 5).

**Table 4:** Prior and adjusted risk for spinal muscular atrophy carrier status when two or three *SMN1* copies are detected by quantitative assays

Ethnicity	Prior risk†	Adjusted risk	
		2 copies	3 copies*
Caucasian	1:35	1:632	1:3500
Ashkenazi Jewish	1:41	1:350	1:4000
Asian	1:53	1:628	1:5000
African American	1:66	1:121	1:3000
Hispanic	1:117	1:1061	1:11000

*The risk estimates were rounded to two significant digits due to approximations in their calculations

†Prior risk is the probability of having any carrier genotype (1+0, 1+1^D^, 2+0 and 2+1^D^).

**Table 5:** Negative predictive value and detection rate by quantitative testing

Ethnicity	Negative predictive value (%)	Detection rate (%)
Caucasian	99.85	94.92
Ashkenazi Jewish	99.76	90.16
Asian	99.86	92.55
African American	99.56	71.12
Hispanic	99.92	90.57

## Discussion

The genetic basis for the unusually high *SMN1* copy number in the African American population is unknown. Previously published data have shown that individuals with higher copy numbers of *SMN1* tend to have fewer copies of *SMN2*.^22^ It has been suggested that this correlation indicates that *SMN2* may have converted to *SMN1*; however, the inverse may also be true. Alleles with multiple copies of *SMN1* may be the ancestral form of the duplication, with conversion to *SMN2* a more recent mutation event. An assessment of *SMN2* copy number is currently underway to understand the *SMN1*/*SMN2* correlation in this sample set.

Prediction of disease phenotype in SMA is complicated by the modifying effects of *SMN2* copy number, as well as other genes such as plastin 3.^23^ Additionally, there is not a completely predictive correlation between phenotype and modifiers. Similar to other autosomal diseases with variable phenotypes, SMA carrier screening results cannot predict the disease phenotype for offspring. Although this study has demonstrated that significant differences exist in SMA carrier frequency between several ethnic groups, an assessment of pregnancy outcomes data for these populations will be needed to determine if disease frequencies or phenotype incidences also vary. More widespread carrier screening will aid in identifying couples at risk of having SMA offspring and allow for appropriate follow-up to answer these questions.

The recent ACMG guideline for SMA carrier screening^17^ recommends that since SMA is present in all populations, carrier testing should be offered to couples regardless of race or ethnicity who are pregnant or considering pregnancy. The results from this study provide precise estimates of allele frequencies and genotypes that vary significantly among ethnic groups. The implications of this work are most significant for Hispanic patients who carry lower risks of *SMN1* mutations and for African American patients who bear increased frequencies of two-copy alleles. These data will facilitate the accurate interpretation of clinical testing results and provide additional information for genetic counselling.
